# High Risk of Depressive Disorders in Patients With Gout: A Nationwide Population-Based Cohort Study: Erratum

**DOI:** 10.1097/01.md.0000484180.59880.52

**Published:** 2016-05-13

In the articles “High Risk of Depressive Disorders in Patients With Gout: A Nationwide Population-Based Cohort Study”,^1^ Dr. Ming-Chia Lin's affiliation was not reported in full. Dr. Lin is affiliated with the E-Da Hospital. The articles have since been corrected online.
